# Community healthcare in Israel: quality indicators 2007-2009

**DOI:** 10.1186/2045-4015-1-3

**Published:** 2012-01-30

**Authors:** Dena H Jaffe, Amir Shmueli, Arie Ben-Yehuda, Ora Paltiel, Ronit Calderon, Arnon D Cohen, Eran Matz, Joseph K Rosenblum, Rachel Wilf-Miron, Orly Manor

**Affiliations:** 1 Program directorate of the National Program for Quality Indicators in Community Healthcare in Israel, Ministry of Health, Israel; 2 Braun School of Public Health and Community Medicine, Hebrew University - Hadassah, POB 12272, Jerusalem, 92210 Israel; 3 Department of Internal Medicine, Hadassah - Hebrew University Medical Center, POB 12000, Jerusalem, 92210 Israel; 4 Clalit Health Services, 101 Arlozorov St., POB 16250, Tel Aviv, 62098 Israel; 5 Leumit Health Fund, 23 Sprinzak St., Tel Aviv, 64738 Israel; 6 Meuhedet Health Fund, 124 Ibn Gvirol St., Tel Aviv, 62038 Israel; 7 Maccabi Healthcare Services, Tel Aviv, 68125 Israel

**Keywords:** Community healthcare, indicators, Israel, National Health Insurance law, quality, QICH

## Abstract

**Background:**

The National Program for Quality Indicators in Community Healthcare in Israel (QICH) was developed to provide policy makers and consumers with information on the quality of community healthcare in Israel. In what follows we present the most recent results of the QICH indicator set for 2009 and an examination of changes that have occurred since 2007.

**Methods:**

Data for 28 quality indicators were collected from all four health plans in Israel for the years 2007-2009. The QICH indicator set examined six areas of healthcare: asthma, cancer screening, cardiovascular health, child health, diabetes and immunizations for older adults.

**Results:**

Dramatic increases in the documentation of anthropometric measures were observed over the measurement period. Documentation of BMI for adolescents and adults increased by 30 percentage points, reaching rates of 61% and 70%, respectively, in 2009. Modest increases (3%-7%) over time were observed for other primary prevention quality measures including immunizations for older adults, cancer screening, anemia screening for young children, and documentation of cardiovascular risks. Overall, rates of recommended care for chronic diseases (asthma, cardiovascular disease and diabetes) increased over time. Changes in rates of quality care for diabetes were varied over the measurement period.

**Conclusions:**

The overall quality of community healthcare in Israel has improved over the past three years. Future research should focus on the adherence to quality indicators in population subgroups and compare the QICH data with those in other countries. In addition, one of the next steps in assessing and further improving healthcare quality in Israel is to relate these process and performance indicators to health outcomes.

## Background

Performance indicators are often used to examine and quantify the various components of healthcare, such as effectiveness of care, safety, timeliness, patient-centeredness, access and efficiency [1]. Comparing indicator results between healthcare systems allows administrators and policy makers to learn "...from the many experiences of others, drawing lessons on how to finance, manage, and organize health care so as to improve health system performance" [2]. The OECD describes its Health Care Quality Indicator Project as an initiative to be used "...to understand why differences exist and what can be done to reduce those differences and improve care in all countries" [3].

In March 2004 the Israel National Institute for Health Policy and Health Services Research, with the support of the Health Council inaugurated the National Program for Quality Indicators in Community Healthcare in Israel (QICH). This program began in 1999 as a research project by Avi Porath, Gad Rabinovitch and Anat Raskin-Segal from Ben-Gurion University. Its success has been, first and foremost, a result of the full support and cooperation of all four health plans between each other and with the program in developing, assessing and publishing the national quality indicators. Many of the QICH indicators are based on definitions from existing international measures, such as those in the Healthcare Effectiveness Data and Information Set (HEDIS) of the National Committee for Quality Assurance (NCQA) in the United States, and with the intention of international comparison. Since QICH's establishment, four national reports on the quality of healthcare in Israel have been published [4-7].

The primary goal of QICH is to provide policy makers and consumers with aggregate information on the quality of community healthcare in Israel in order to monitor and improve the medical care system. The QICH indicator set is based on national and international medical guidelines that reflect the current scientific evidence. Indicators are well-defined, measurable items agreed upon by all stakeholders including health plans and professional organizations. Data are systematically collected for the entire population of Israel from all four health plans to create national-level healthcare quality indicators that are publically reported. In what follows is an overview of the results of the most recent QICH report with specific attention to changes over time and implications to the primary healthcare system in Israel.

## Methods

### Data source

Data for quality indicators are collected independently by each of the four health plans for their insured population. Each health plan maintains individual-level electronic data records that include medical appointments, procedures, laboratory test results and pharmacy claims. Quality indicators sent by each of the four health plans are aggregated and these values (numerators and denominators) are used to calculate national rates. Coordination, data compilation, validation and quality assessment and analysis of the national indicators are performed by the program directorate.

### Population

All Israeli residents are included in the data set. The quality indicators presented represent the time period 2007 through 2009. Electronic records were unavailable for a small percent of the population (< 1%). In addition, individuals whose membership in the plan was less than the full calendar year were excluded from the calculations. In 2009, for example, approximately 106,800 people or 1.4% of the insured population from the previous year switched healthcare plan [8] and did not have the required data for a complete measurement year. It is noteworthy that soldiers, largely comprised of individuals aged 18-21 years, are not covered by the health plans since their medical care is delivered by the military. Aside from these exceptions, the report includes the entirety of Israel's population, approximately seven million people.

### Quality indicators and data

**Figure 1:**
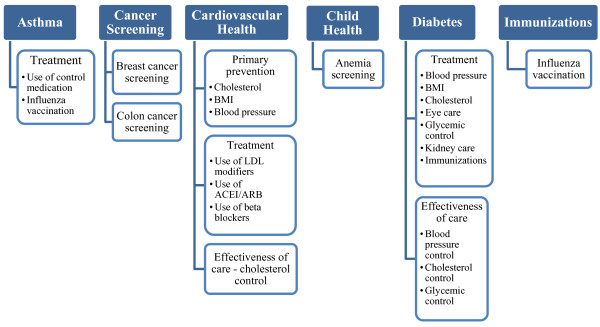
**Quality indicators of the National Program for Quality Indicators in Community Healthcare in Israel, 2007-2009**.

Quality indicators were created with the consensus of the four health plans and Israeli medical associations. The HEDIS indicator set was used as a guide for the QICH indicator definitions. The QICH indicator set reflects a careful selection process that assessed the feasibility of production and the applicability and significance of the indicators for the healthcare system in Israel. Data were collected according to gender and age group. Six areas of healthcare - asthma, cancer screening, immunizations for older adults, child health, cardiovascular health and diabetes were used to assess the quality of community healthcare in Israel. Twenty-eight QICH indicators are presented for the years 2007-2009 (Figure 1). Identification of individuals with asthma and diabetes was according to pharmacy claims. Patients with cardiovascular disease were identified according to cardiac intervention - bypass surgery or coronary angioplasty. It should be noted, that these procedures were the only available diagnostic indicators of cardiovascular disease accessible to all health plans and therefore the number of patients included in this sub-population reflect a small percentage of individuals with the disease. Immunization status was established using pharmacy claims (i.e., vaccine purchase).

Data quality assessment as well as performance audits were carried out in three stages: by each health plan, by the QICH program directorate and by an accredited external auditor. Data checks that included logical tests for negative numbers, unit tests, and valid numerator and denominator entries as well as tests examining subgroups and changes over time were performed by each health plan internally and by the QICH program directorate. An external auditor conducted a procedural audit for each health plan as well as the for the QICH directorate. A system-oriented approach to examining the proper functioning and management of the program was performed and documented in comprehensive performance reports.

Rate differences were calculated as the absolute difference in percentage points between the rate in 2007 and 2009.

## Results

Table 1 provides details for the definition and calculation of each of the 28 quality indicators, the target population, measurement period and the relevant data sources.

**Table 1:** Definitions and data sources of the QICH indicator set, 2007-2009.

Healthcare quality indicators	Domain	Data source^a^	Target population
**Asthma**			
Use of appropriate control medication for individuals with persistent asthma^b^ Percentage of individuals with persistent asthma who purchased at least three asthma control medications in three different months during the measurement year	Treatment	Pharmacy claims	5-56 years

Influenza vaccination for individuals with persistent asthma^b^ Percentage of individuals with persistent asthma who received the influenza vaccination during the winter months of the measurement year (plus two months of subsequent year)	Treatment	Pharmacy claims	5-56 years
			
**Cancer screening**			
Breast cancer screening: Mammography Percentage of women who had a mammogram during the past two years	Primary prevention	Test records	51-74 years
Colon cancer screening: Fecal occult blood test Percentage of individuals who had a fecal occult blood test during the measurement year^c^	Primary prevention	Laboratory results	50-74 years

			
**Cardiovascular health**			
Blood pressure documentation Percentage of individuals with blood pressure documentation	Primary prevention	Medical records	20-54 years - documentation during past five years; 55-74 years - documentation during measurement year

Blood pressure assessment Percentage of individuals with adequate blood pressure control (≤ 140/90 mmHg)	Primary prevention	Medical records	20-54 years - documentation during past five years; 55-74 years - documentation during measurement year

Body mass index (BMI) documentation • Percentage of individuals with BMI documentation - adolescents • Percentage of individuals with BMI documentation - adults	Primary prevention	Medical records	14-18 years - documentation during past three years; 20-64 years - documentation during past five years; 65-74 years - documentation of weight during the measurement year and height during the past five years
Cholesterol documentation Percentage of individuals with cholesterol documentation	Primary prevention	Laboratory results	35-54 years - documentation during past five years; 55-74 years - documentation during measurement year
Cholesterol assessment Percentage of individuals with adequate low-density lipoprotein cholesterol control (≤ 130 mg/dL)	Primary prevention	Laboratory results	35-54 years - documentation during past five years; 55-74 years - documentation during measurement year

Treatment with statins and other low-density lipoprotein (LDL) modifying medications following a coronary intervention^d^ Percentage of individuals following a coronary event who purchased at least three LDL-modifying medications in three different months during the measurement year	Treatment	Pharmacy claims, Hospital procedures	35-74 years

Treatment with angiotensin-converting enzyme inhibitors (ACEI) or angiotensin receptor blockers (ARB) following a coronary intervention^d^ Percentage of individuals following a coronary event who purchased at least three ACEI/ARB medications in three different months during the measurement year	Treatment	Pharmacy claims, Hospital procedures	35-74 years

Treatment with beta blockers following a coronary intervention^d^ Percentage of individuals following a coronary event who purchased at least three ACEI/ARB medications in three different months during the measurement year	Treatment	Pharmacy claims, Hospital procedures	35-74 years

Cholesterol assessment following a coronary intervention^d^ Percentage of individuals following a coronary event with adequate cholesterol control (≤ 100 mg/dL) during the measurement year	Effectiveness of care	Laboratory results, Hospital procedures	35-74 years
			
**Child health**			
Anemia screening in infants Percentage of children who had a hemoglobin test during the measurement year	Primary prevention	Laboratory results	1 year olds
			
**Diabetes**			

Blood pressure documentation Percentage of patients with diabetes mellitus with blood pressure documentation during the measurement year	Treatment	Laboratory results	18+ years

Body mass index (BMI) documentation Percentage of patients with diabetes mellitus with BMI documentation	Treatment	Medical records	18+ years - documentation of weight during the measurement year and height over the past five years

Cholesterol documentation Percentage of patients with diabetes mellitus with cholesterol documentation during the measurement year	Treatment	Laboratory results	All ages

Eye care documentation Percentage of patients with diabetes mellitus with documentation of an eye examination during the measurement year	Treatment	Medical records	All ages

Glycemic control documentation Percentage of patients with diabetes mellitus with glycemic control (HbA1c) documentation during the measurement year	Treatment	Laboratory results	All ages

Kidney function documentation Percentage of patients with diabetes mellitus with microalbuminurea documentation during the measurement year	Treatment	Laboratory results	All ages

Influenza vaccination Percentage of patients with diabetes mellitus who received the influenza vaccination during the winter months of the measurement year (plus two months of subsequent year)	Treatment	Pharmacy claims	5+ years

Blood pressure assessment Percentage of patients with diabetes mellitus with adequate blood pressure control (≤ 130/80 mmHg) during the measurement year	Effectiveness of care	Medical records	18+ years

Cholesterol assessment Percentage of patients with diabetes mellitus with adequate low-density lipoprotein cholesterol control (≤ 100 mg/dL) during the measurement year	Effectiveness of care	Laboratory results	All ages

Glycemic control assessment Percentage of diabetics with adequate glycemic control (HbA1c ≤ 7%) during the measurement year	Effectiveness of care	Laboratory results	All ages

Assessment of inadequate glycemic control Percentage of patients with diabetes mellitus with inadequate glycemic control (HbA1c > 9%) during the measurement year	Effectiveness of care	Laboratory results	All ages
Treatment with insulin for individuals with inadequate glycemic control Percentage of patients with diabetes mellitus with inadequate glycemic control (HbA1c > 9%) and purchased insulin at least once during the measurement year	Effectiveness of care	Laboratory results, Pharmacy claims	All ages
			
**Immunizations for older adults**			
Influenza vaccination for older adults Percentage of older adults who received the influenza vaccination during the winter months of the measurement year (plus two months of subsequent year)	Primary prevention	Pharmacy claims	65+ years

BMI, body mass index; LDL, low density lipoproteins; ACEI, angiotensin-converting enzyme inhibitors; ARB, angiotensin receptor blockers; HbA1c, glycated hemoglobin; QICH, National Program for Quality Indicators in Community Healthcare in Israel

^a^All data were collected from each MCO's electronic records. Numerator and denominator data for each measure were collected by the National QICH project to create the national rate.

^b ^Persistent asthma is defined by asthma medication purchases. These medications include control medication (immunomodulators, inhaled corticosteroids, leukotriene modifiers, long-acting beta-2 agonists, methylxanthines, mast cell stabilizers), as well as relief medications (short-acting beta-2 agonists, anticholinergics).

^c ^Excluding those who underwent a colonoscopy during the past five years.

^d^A coronary intervention is defined as having undergone coronary artery bypass surgery or cardiac catheterization during the past five years.

Table 2 presents the changes in the quality indicators over the period 2007-2009. Both absolute and relative changes are provided in the table.

**Table 2:** Rates and change over time in the QICH indicator set, 2007-2009.

Healthcare quality indicators	Denominator population^a^	Rates			Rate difference^b^	Rate change (%)^b^
				
		2007	2008	2009		
**Asthma**						
Treatment: influenza vaccination for individuals with persistent asthma	51,931	29.1%	31.6%	40.0%	+10.9	37.5
Treatment: Use of appropriate control medication for individuals with persistent asthma	51,931	76.2%	78.1%	79.7%	+3.5	4.6
**Cancer**						
Primary prevention: breast cancer screening - mammography	695,621	60.7%	64.7%	67.7%	+7.0	11.5
Primary prevention: colon cancer screening - fecal occult blood test	1,078,021	22.1%	24.4%	27.4%	+5.3	24.0
**Cardiovascular health**						
Primary prevention: body mass index documentation (adolescents)	511,374	27.9%	46.3%	60.8%	+32.9	117.9
Primary prevention: body mass index documentation (adults)	3,719,937	43.9%	56.6%	69.7%	+25.8	58.8
Primary prevention: blood pressure documentation	3,755,235	72.8%	78.3%	83.5%	+10.7	14.7
Effectiveness of care: cholesterol assessment of adequate control following cardiac intervention	56,726	68.6%	71.4%	72.1%	+3.5	5.1
Treatment: treatment with ACEI/ARB following cardiac intervention	65,299	63.1%	64.7%	66.4%	+3.3	5.2
Primary prevention: cholesterol documentation	2,575,726	77.4%	79.0%	80.5%	+3.1	4.0
Primary prevention: cholesterol assessment of adequate control	2,072,458	68.9%	70.9%	71.7%	+2.8	4.1
Treatment: treatment with beta blockers following cardiac intervention	65,299	68.4%	67.9%	70.2%	+1.8	2.6
Primary prevention: blood pressure assessment of adequate control	3,135,531	92.9%	93.6%	94.1%	+1.2	1.3
Treatment: treatment with statins and other LDL modifying medications following a cardiac intervention	65,299	84.2%	83.8%	84.6%	+0.4	0.5
**Child health**						
Primary prevention: anemia screening for infants	151,741	66.4%	70.5%	73.5%	+7.1	10.7
**Diabetes**						
Treatment: BMI documentation	320,275	74.4%	81.3%	83.6%	+9.2	12.4
Effectiveness of care: treatment of inadequate glycemic control	39,514	44.8%	48.8%	53.1%	+8.2	18.5
Treatment: influenza vaccination	332,706	47.1%	51.4%	55.0%	+7.9	16.8
Effectiveness of care: cholesterol - adequate control	300,885	60.3%	63.7%	65.6%	+5.3	8.8
Treatment: kidney function documentation	332,854	71.3%	70.4%	74.3%	+3.0	4.2
Treatment: blood pressure documentation	330,437	90.0%	91.9%	91.9%	+1.9	2.1
Effectiveness of care: blood pressure - adequate control	303,710	67.0%	68.5%	68.6%	+1.6	2.4
Treatment: eye care documentation	332,854	63.0%	63.5%	64.3%	+1.3	2.1
Treatment: glycemic control documentation	332,854	91.7%	91.6%	92.3%	+0.6	0.7
Effectiveness of care: glycemic control - inadequate control^c^	307,244	13.3%	13.5%	12.9%	-0.4	-3.0
Treatment: cholesterol documentation	332,854	90.9%	90.3%	90.4%	-0.5	-0.6
Effectiveness of care: glycemic control - adequate control	307,244	49.4%	47.9%	48.0%	-1.4	-2.8
**Immunizations for older adults**						
Primary prevention: influenza vaccination for older adults	709,755	51.9%	55.3%	56.7%	+4.8	9.2

BMI, body mass index; LDL, low density lipoproteins; ACEI, angiotensin-converting enzyme inhibitors; ARB, angiotensin receptor blockers; QICH, National Program for Quality Indicators in Community Healthcare in Israel

^a^The denominator population are all eligible individuals in 2009.

^b^The numbers represent absolute differences in percentage points from 2007 to 2009 or changes relative to 2007.

^c^Decreased rates indicates a greater quality of care.

### Asthma

Rates of appropriate use of asthma control medication for individuals with persistent asthma increased over the three-year period and influenza immunization for this group increased to a rate of 40% by 2009 (absolute increase of 11%).

### Cancer screening

In 2009, the mammography rate for women over 51-74 years reached 68% and fecal occult blood test rate for colon cancer (excluding those who underwent a colonoscopy during the past five years) for individuals aged 50-74 years was 27%. Absolute rate increases of 5%-7% were observed for both types of cancer screening over the three-year measurement period.

### Cardiovascular health

Quality measures of primary prevention of cardiovascular disease, which included BMI (adults) and blood pressure documentation, increased to rates of 70% and 84%, respectively, for the 3.8 million Israeli adult target population aged 20-74 years. Rates of cholesterol documentation and control were 81% and 72%, respectively, for Israeli adults, ages 35-74 years.

Measures of appropriate treatment and effectiveness of treatment improved over the measurement period. For example, rates of use of medications, such as beta blockers and statins, for individuals following a coronary heart event were over 70%. The rate of appropriate control of cholesterol for cardiac heart patients was 72% in 2009; this represented an absolute change during the three-year period of +3.5%.

Over the three-year measurement period, positive changes in rates for cardiovascular care were observed for all aspects of treatment - prevention, care and effectiveness of care. In particular, substantial increases were observed for documentation rates of body mass index for adolescents and adults. For adolescents, rates of documentation of height and weight increased dramatically from 28% in 2007 to 61% in 2009, while for adults, these rates rose 26 percentage points over the same time period.

### Child health

An indicator of quality of healthcare for primary prevention for children was examined. In 2009, 74% of children 1-year of age underwent anemia screening - an absolute increase of 7% from 2007.

### Diabetes

Quality indicators for diabetes assessed both the provision and the effectiveness of care. Patients with diabetics mellitus received basic recommended care, such as glucose, blood pressure and cholesterol assessment (> 90%), BMI documentation (84%), kidney function testing (74%) and eye care documentation (64%). Only about half the population of diabetics received an influenza immunization in the winter season. Individuals with diabetes mellitus who attained adequate glycemic control (HbA1c ≤ 7%) decreased slightly over the measurement period (49% to 48%) and remained stable among those with poor glycemic control (13%). The percent of individuals with diabetes mellitus who reached pre-specified cholesterol and blood pressure targets showed an increase of 5.3% and 1.6% respectively.

### Immunizations for older adults

The rates of influenza vaccination, a quality indicator of preventive care among Israelis 65+ years, increased over the measurement period from 52% to 57%.

## Discussion

Overall, the quality in community healthcare in Israel as measured by the QICH indicator set has improved over the past three years. Increased levels of quality of care were observed across almost all categories of health as well as type of care - primary prevention, treatment and effectiveness of care.

It should be noted that there are no set target standards for quality measures in community healthcare in Israel. Nevertheless, results from similar international indicators are used as benchmarks for comparison and learning tools for quality improvement [9]. Moreover, the QICH indicator set is based on HEDIS definitions, which strengthens the validity of the measures and encourages comparison. International comparisons are complicated, however, by numerous factors such as differences between healthcare structures and measure definitions [10,11]. For example, variations exist between measure specifications (e.g., age limits), disease definition (e.g., based on physician diagnosis, hospital discharge data and/or pharmacy records), reporting period and data collection methods (e.g., self-reported data versus physician documentation). The HEDIS program, for instance, collects data from three different healthcare provider types - Medicaid, Medicare and commercial insurers. Differences between countries may also arise from variations in guidelines of care, type and mode of care and resources allocated for care. For example, Israel's quality indicators are used as healthcare surveillance tools while the Quality and Outcomes Framework (QOF) in England is a performance-based indicator system that rewards good practice.

### Quality indicators for primary prevention

The most dramatic increases in the QICH indicator set 2007-2009 were observed for documentation of anthropometric measures. Documentation rates for height and weight for the calculation of BMI among adolescents and adults increased by approximately 30% over this time period to rates of 61% and 70%, respectively. Rates in Israel are high relative to adult BMI screening rates in the United States (e.g., 35% for Medicaid and 41% for commercially insured populations) [12].

Moderate increases over time were observed for other primary prevention QICH measures, including immunizations for older adults, cancer screening, anemia screening and documentation of cardiovascular risk factors such as blood pressure and cholesterol. Comparison of these measures with those in other healthcare systems reveals wide variations in rates between countries. For example, the rate of influenza immunization for older adults in Israel in 2009 was 57%, similar to that of Ireland and Luxembourg and in contrast to higher rates in Mexico (88%) and France (71%) and lower rates in Finland (43%) and Hungary (32%) [13]. Mammography screening rates in Israel were similar to those of the OECD (68% versus 62%, respectively), although substantial variations exist between countries - high mammography rates in Finland (86%) and Ireland (78%) and low rates in France (47%) and Japan (24%) [14]. Anemia screening is not routinely conducted in most Western countries thereby preventing comparisons. Recently released guidelines of the American Academy of Pediatrics recommend universal screening for anemia for children 12 months of age to determine hemoglobin concentrations for the assessment of iron deficiency [15]. This step may generate future comparisons. Finally, documentation of cardiovascular risk factors, such as cholesterol and blood pressure levels, is essential for evaluating an individual's risk of heart disease [16]. Documentation rates of these factors have increased between 4%-15% from 2007 to 2009 and likely reflect the increased attention of the healthcare system to cardiovascular risks.

### Quality indicators for chronic disease

Rates of recommended care for three chronic conditions - asthma, cardiovascular disease and diabetes improved over the measurement period and are nearing those in other Western countries [12,17]. For example, appropriate use of control medication for individuals with persistent asthma improved by four percentage points between 2007 and 2009; these rates remain lower than a similar HEDIS quality indicator (91%) [12].

For indicators assessing care of patients with cardiovascular disease, at least 70% of Israelis following a coronary intervention received the recommended treatment with statins, beta blockers or ACEI/ARBs. For patients following a coronary intervention, similarities between Israel and England were observed for treatment with beta blockers (Israel = 70%; England = 74%) but differed for treatment with ACEI/ARB (Israel = 69%; England = 89%) [17]. Notably the QICH indicator set defined coronary intervention as persons who underwent bypass surgery or coronary angioplasty in the past five years, while the QOF was based on patients who had a history of myocardial infarction since April 2003.

Treatment and care for diabetes in Israel has moved in a positive direction over the three year measurement period, although changes in rates for indicators of glycemic control were relatively small and even negative. Seven indicators of adherence to recommended treatment of diabetes mellitus were assessed. For the measurement period 2007-2009, higher rates (≥ 74%) of appropriate care were noted in four measures - documentation of BMI, kidney function, glycemic control and cholesterol, and relatively moderate rates (55%-74%) were observed in the remaining three areas - documentation of blood pressure and eye care and influenza immunization. The observed trends for adequate glycemic control (< 7%) are noteworthy. The results suggest that the practice of tight glycemic control may be changing among primary care physicians from a generalized HbA1c goal to an individualized target. Indeed, recent studies have shown that glycemic control for all patients with Type 2 diabetes may not provide the ultimate benefit [18]. One of the current goals of the QICH program is to revise this measure and determine sub-group-specific HbA1c goals. Decreases, albeit slight, in inadequate glycemic control (> 9%) indicate improvements in diabetes care. Further monitoring is necessary to identify long-term trends. Comparison with similar indicators and age groups from the HEDIS and England reveal moderate variations between countries and measures [12,17]. For example, documentation of HbA1c among adults ranged from 92% in Israel, 97% in the England and 89% in the United States [12,17]. Similarly, rates of microalbuminurea testing (kidney function) varied from 74% in Israel, 89% in the England and 84% in the United States [12,17].

### Quality indicators and policy

Improvements in quality of care have been noted previously in Israel [19]. In this recent study, the authors hypothesize that quality improvements may be a result of the feedback from the measurement process itself. This theory relates to the quality improvement maxim that "efforts to improve quality require efforts to measure it" [20]. To be sure, during the past decade of quality assessment of healthcare in Israel, health plans have implemented strategies for improving care and access to services as well as encouraged physician adherence to guidelines [21,22]. The dramatic increases in documentation of anthropometric measures signify a targeted approach by health plans to document a neglected area of care and signal policy changes that have permeated all segments of the healthcare system. In light of these observations, expected trajectories of existing quality indicators are likely to attenuate over the next decade.

It should be noted, however, that incentives may account for lower rates of healthcare quality in certain areas in comparison to England's pay-for-performance system since the Israeli healthcare system is not a pay-for-performance system and physicians do not receive direct benefits for increased performance. In order to properly assess and compare the quality of Israel's healthcare system with that of a pay-for-service, outcome-based indicators and sufficient follow-up time are necessary.

Policy implications and future directions of this program mirror that of other international efforts [23,24]. Quality indicators must undergo constant evaluation of whether they accurately capture the care that is being provided as well as their impact on health outcomes [24]. Examination of the adverse or beneficial changes in health outcomes is an essential tool in the evaluation process, since change alone in quality of care does not necessarily correspond to improvements in patient health. Composite indicators of health are also integral to disease management and future indicator sets should comprise such measures.

Assessment of quality indicators should comprise a more refined evaluation of subgroups and at-risk populations as well as changes over time in their quality care. In a recent report by Hussey et al. [10], similar disparities in socio-economic status (SES) in healthcare quality indicators were observed for four countries with different health systems and populations. The authors note that their study underscores the need to examine the impact of internal and external factors on the healthcare system (and health inequalities), such as changing healthcare patterns (internal) and targeted prevention programs (external). The proxy SES variable available to the QICH data set (data not shown) is of questionable utility and a proper assessment of the effect of SES on quality care in Israel is needed.

Finally, The QICH program has brought about substantial positive changes in the quality of healthcare data in Israel. An improved data platform that captures a larger share of the healthcare delivery system is necessary for the further assessment of continuity of care and other healthcare quality issues. Policymakers should promote the continued interface between the four Israeli health plans and support communication with other healthcare providers, such as for residential, early childhood and elderly care facilities. Interchange between hospital and primary care is vital to improved healthcare quality such as effectiveness and efficiency of care and care coordination. This type of improved framework requires policy changes and even incentives to allow for information exchange and data flow within the confines of privacy and confidentiality.

### Strengths and limitations

Israel is one of the only countries with a systematic and comprehensive evaluation program assessing the quality of the community healthcare at a national level. The QICH indicator set represents a unique and essential tool in healthcare. First, quality indicators are important to assess adherence of the community healthcare system to recommended guidelines that include preventive care. Increased use of preventive healthcare services is considered essential for reducing the clinical preventable burden as well as being cost effective [25]. Second, electronic health records - a mainstay of the medical system in Israel for over a decade, allow for the assessment of historical as well as current healthcare measurements. Third, all residents have unique identifying numbers that allow for linking external medical records, such as those for hospital procedures. Fourth, the program is a combined effort of all four health plan providers, who have, among other things, standardized disease registries and confirmed their commitment to treatment guidelines.

These quality indicators are not without limitations. Quality of community healthcare is anchored in its governing bodies and provider system and is susceptible to the constraints and shortcomings of each. International comparisons with similar measures are subject to the diversity of policy, culture and resources that directly and indirectly affect the structure, performance and outcome of healthcare [26]. In Israel, health plans are restricted by the availability of certain types of healthcare data, such as hospital discharge data and inpatient diagnoses. For example, patients with heart disease are identified using reimbursement codes for bypass surgery or coronary angioplasty even though this sub-population represents only about 10% of the patients with cardiovascular disease. The validity of the QICH indicator set must also be qualified. Apart from laboratory results, which produce objective and non-biased values, blood pressure measurements, for example, which require human assessment and documentation, vary between testers and may be subject to dishonest reporting. Lastly, regulatory restrictions of demographic information, including socio-economic status, limit the development of a complete assessment of quality across all categories of health and for various subgroups. Case-mix adjustments or stratification according to demographic or socio-economic characteristics may allow for a clearer understanding of the quality of health care in Israel in order to target gaps in care and identify and learn from successes.

## Conclusions

The overall quality of community health care in Israel has improved over the past three years. Comparisons with similar international quality indicators suggest that community healthcare in Israel is on par with other Western countries. The results presented in this study illustrate that the current state of health care in Israel is that of improving quality of community healthcare. One of the next steps in assessing and improving healthcare quality in Israel is to relate these process and performance indicators to health outcomes.

## Abbreviations

ACEI: angiotensin-converting enzyme inhibitors; ARB: angiotensin receptor blockers; BMI: body mass index; HbA1c: glycated hemoglobin; LDL: low density lipoproteins; QICH: Quality Indicators in Community Healthcare; QOF: Quality and Outcomes Framework; NIH: National Health Insurance; SES: socio-economic status.

## Competing interests

No financial or non-financial competing interests are declared. All results are derived from national-level data that were not influenced by or reflect any one institution.

## Authors' contributions

DHJ, OM, AS, AB-Y OP and RC drafted the article and DHJ wrote the manuscript. All authors reviewed the draft manuscript and read and approved the final manuscript.

## Authors' information

DHJ is an epidemiologist at the School of Public Health, Hebrew University - Hadassah and the coordinator of the National Program for Quality Indicators in Community Healthcare in Israel.

OM is a professor of statistics, head of the School of Public Health, Hebrew University - Hadassah and is the director of the National Program for Quality Indicators in Community Healthcare in Israel.

AS is a professor of health economics at the School of Public Health, Hebrew University - Hadassah and is on the directorate of the National Program for Quality Indicators in Community Healthcare in Israel.

AB-Y is a professor of internal medicine, head of the department of internal medicine at the Hadassah Medical Center and is on the directorate of the National Program for Quality Indicators in Community Healthcare in Israel.

OP is a professor of hematology at the Hadassah Medical Center, epidemiologist at the School of Public Health, Hebrew University - Hadassah and is on the directorate of the National Program for Quality Indicators in Community Healthcare in Israel.

RC is a physician and epidemiologist at the School of Public Health, Hebrew University - Hadassah and is on the directorate of the National Program for Quality Indicators in Community Healthcare in Israel.

ADC is a physician and Director of the Department of Quality Measures and Research, Chief Physician Office at Clalit Health Services and is on the steering committee of the National Program for Quality Indicators in Community Healthcare in Israel.

JKR is a physician and CIO of the Medical Division of the Meuhedet Health Fund and is on the steering committee of the National Program for Quality Indicators in Community Healthcare in Israel.

EM is a physician and Director of the Division of Clinical Medicine at the Leumit Health Fund and is on the steering committee of the National Program for Quality Indicators in Community Healthcare in Israel.

RW-M is a physician and Director of Quality Management at Maccabi Healthcare Services and is on the steering committee of the National Program for Quality Indicators in Community Healthcare in Israel.
